# Outcomes after ischemic stroke for dual-eligible Medicare-Medicaid beneficiaries in the United States

**DOI:** 10.1371/journal.pone.0292546

**Published:** 2023-10-05

**Authors:** Erica C. Leifheit, Yun Wang, Larry B. Goldstein, Judith H. Lichtman

**Affiliations:** 1 Department of Chronic Disease Epidemiology, Yale School of Public Health, New Haven, Connecticut, United States of America; 2 Center for Outcomes Research and Evaluation, Yale-New Haven Hospital, New Haven, Connecticut, United States of America; 3 Department of Internal Medicine, Section of Cardiovascular Medicine, Yale School of Medicine, New Haven, Connecticut, United States of America; 4 University of Kentucky College of Medicine and Kentucky Neuroscience Institute, Lexington, Kentucky, United States of America; Harbor-UCLA Research and Education Institute: The Lundquist Institute, UNITED STATES

## Abstract

**Background:**

Medicaid serves as a safety net for low-income US Medicare beneficiaries with limited assets. Approximately 7.7 million Americans aged ≥65 years rely on a combination of Medicare and Medicaid to obtain critical medical services, yet little is known about whether these patients have worse outcomes after stroke than patients with Medicare alone. We compared geographic patterns in dual Medicare-Medicaid eligibility and ischemic stroke hospitalizations and examined whether these dual-eligible beneficiaries had worse post-stroke outcomes than those with Medicare alone.

**Methods:**

We identified fee-for-service Medicare beneficiaries aged ≥65 years who were discharged from US acute-care hospitals with a principal diagnosis of ischemic stroke in 2014. Medicare beneficiaries with ≥1 month of Medicaid coverage were considered dual eligible. We mapped risk-standardized stroke hospitalization rates and percentages of beneficiaries with dual eligibility. Mixed models and Cox regression were used to evaluate relationships between dual-eligible status and outcomes up to 1 year after stroke, adjusting for demographic and clinical factors.

**Results:**

At the national level, 12.5% of beneficiaries were dual eligible. Dual-eligible rates were highest in Maine, Alaska, and the southern half of the United States, whereas stroke hospitalization rates were highest in the South and parts of the Midwest (Pearson’s r = 0.469, p<0.001). Among 254,902 patients hospitalized for stroke, 17.4% were dual eligible. In adjusted analyses, dual-eligible patients had greater risk of all-cause readmission within 30 days (hazard ratio 1.06, 95% confidence interval [CI] 1.03–1.09) and 1 year (hazard ratio 1.03, 95% CI 1.02–1.05) and had greater odds of death within 1 year (odds ratio 1.20, 95% CI 1.17–1.23) when compared with Medicare-only patients; there was no difference in in-hospital or 30-day mortality.

**Conclusion:**

Dual-eligible stroke patients had higher readmissions and long-term mortality than other patients, even after comorbidity adjustment. A better understanding of the factors contributing to these poorer outcomes is needed.

## Introduction

There were 7.7 million US adults aged 65 years or older who relied on both Medicaid and Medicare for their healthcare coverage in 2019 [1]. These dual Medicare-Medicaid-eligible beneficiaries are among the most socioeconomically vulnerable in the US healthcare system, and their numbers have increased by 2.4 million since 2006 [1]. Dual eligibility is a marker of low income and limited financial assets, but it also identifies Medicare beneficiaries receiving additional financial assistance with healthcare costs. We previously noted worse outcomes for dual-eligible versus Medicare-only beneficiaries undergoing carotid revascularization [2], and an analysis of all fee-for-service beneficiaries showed persistent disparities in all-cause hospitalizations and mortality by dual-eligible status from 2004 to 2017 [3]. National data on dual-eligible stroke patients, however, are limited. Accordingly, we compared geographic patterns in dual eligibility and ischemic stroke hospitalizations among US Medicare beneficiaries and evaluated whether dual-eligible stroke patients had worse short-term and long-term outcomes than those with Medicare alone.

## Methods

### Study population

The study included all US adults aged 65 years or older who were enrolled in fee-for-service Medicare in 2014. We calculated beneficiary-years of fee-for-service enrollment to account for changes in enrollment or death, and we linked these data to Medicare inpatient claims to identify patients discharged from acute-care hospitals with a principal diagnosis of ischemic stroke (*International Classification of Diseases*, *Ninth Revision* codes 433, 434, or 436). We used 2013–2014 data to obtain medical history for the year prior to the index stroke and 2014–2015 data to provide outcomes up to 1 year. The institutional review board at Yale University approved the study and waived informed consent for these analyses.

### Patient characteristics

Patients were considered dual eligible if they were enrolled in both Medicaid and Medicare for at least 1 month during the follow-up period [2–4]. Additional patient demographic information included age, sex, race-ethnicity, and ZIP code of residence. Clinical variables (Table 1) were identified using secondary diagnoses from the index admission and inpatient claims from the previous year, based on the Hierarchical Condition Categories [5].

**Table 1:** Patient characteristics by dual Medicare-Medicaid-eligible status.

	Dual eligible (n = 44,476)	Medicare only (n = 210,426)
**Demographic characteristics, n (%)**		
Age, mean ± standard deviation, y	78.3 ± 8.7	78.9 ± 8.6
Women	28642 (64.4)	106664 (50.7)
Race		
White	28292 (63.6)	185654 (88.2)
Black	9683 (21.8)	17452 (8.3)
All Other	6501 (14.6)	7320 (3.5)
**Medical history/comorbidities, n (%)**		
Congestive heart failure	4170 (9.4)	15475 (7.4)
Prior myocardial infarction	759 (1.7)	3274 (1.6)
Unstable angina	463 (1.0)	1716 (0.8)
Chronic atherosclerosis	12585 (28.3)	63285 (30.1)
Hypertension	33583 (75.5)	156296 (74.3)
Diabetes	17904 (40.3)	66691 (31.7)
Peripheral vascular disease	1902 (4.3)	7919 (3.8)
Prior stroke	2996 (6.7)	12252 (5.8)
Cerebrovascular disease	5585 (12.6)	23003 (10.9)
Renal failure	4381 (9.9)	17252 (8.2)
Chronic obstructive pulmonary disease	7944 (17.9)	27528 (13.1)
Pneumonia	3511 (7.9)	11656 (5.5)
Respiratory failure	1524 (3.4)	5445 (2.6)
Cancer	2347 (5.3)	12779 (6.1)
Protein-calorie malnutrition	2901 (6.5)	8673 (4.1)
Functional disability	3000 (6.7)	9737 (4.6)
Dementia	4794 (10.8)	15382 (7.3)
Depression	3497 (7.9)	13947 (6.6)
Other psychiatric disorder	1824 (4.1)	4456 (2.1)
Trauma in past year	2066 (4.6)	9197 (4.4)
Chronic liver disease	458 (1.0)	1218 (0.6)

### Outcomes

Outcomes included in-hospital, 30-day, and 1-year all-cause mortality (measured from the index admission date); and 30-day and 1-year all-cause readmission (measured from the discharge date). We also assessed discharge disposition, length of stay, and Medicare payment for the index hospitalization.

### Statistical analysis

We compared patient demographic characteristics, clinical factors, and observed outcomes by dual-eligible status. We fit a spatial mixed model with a Poisson distribution and county-specific random intercepts for stroke hospitalizations as a function of age, sex, race-ethnicity, and Medicare Part B coverage and accounting for between-county geographic differences. The model included the county-specific number of beneficiaries as an offset and a spherical covariate structure to account for spatial autocorrelation, and it was used to calculate the risk-standardized rate of stroke hospitalization for each county. For both county-level risk-standardized stroke hospitalization and percentage of beneficiaries with dual eligibility, we accounted for county neighboring group effects and obtained smoothed rates by fitting a local linear regression to the risk-standardized rates/percentages as a function of a county’s latitude and longitude, weighted by the county-specific Medicare population. Counties or county-equivalents were grouped into 25 quantiles based on their rates and mapped according to a gradient from green to red (lowest to highest rate). We calculated the weighted Pearson correlation coefficient to quantify the linear relationship between the county-level risk-standardized rates of stroke hospitalization and percentages of dual-eligible beneficiaries.

We fit mixed models with a logit link function to evaluate the relationship between dual-eligible status and in-hospital, 30-day, and 1-year all-cause mortality. For 30-day and 1-year all-cause readmission, we fit Cox proportional hazards models that censored for change in Medicare enrollment and accounted for death as a competing risk using the Fine and Gray method [6]. We used the Schoenfeld residuals to verify the proportional hazards assumption for these models [7]. All models adjusted for age, sex, race-ethnicity, and the following clinical variables: congestive heart failure, prior myocardial infarction, unstable angina, chronic atherosclerosis, hypertension, diabetes, peripheral vascular disease, prior stroke, cerebrovascular disease, renal failure, chronic obstructive pulmonary disease, pneumonia, respiratory failure, cancer, protein-calorie malnutrition, functional disability, dementia, depression, other psychiatric disorder, trauma in the past year, and chronic liver disease.

Analyses were conducted using SAS v9.4 (SAS Institute, Cary, North Carolina). Statistical tests used a two-sided α of 0.05.

## Results

### Rates of dual eligibility and stroke hospitalization

**Fig 1:**
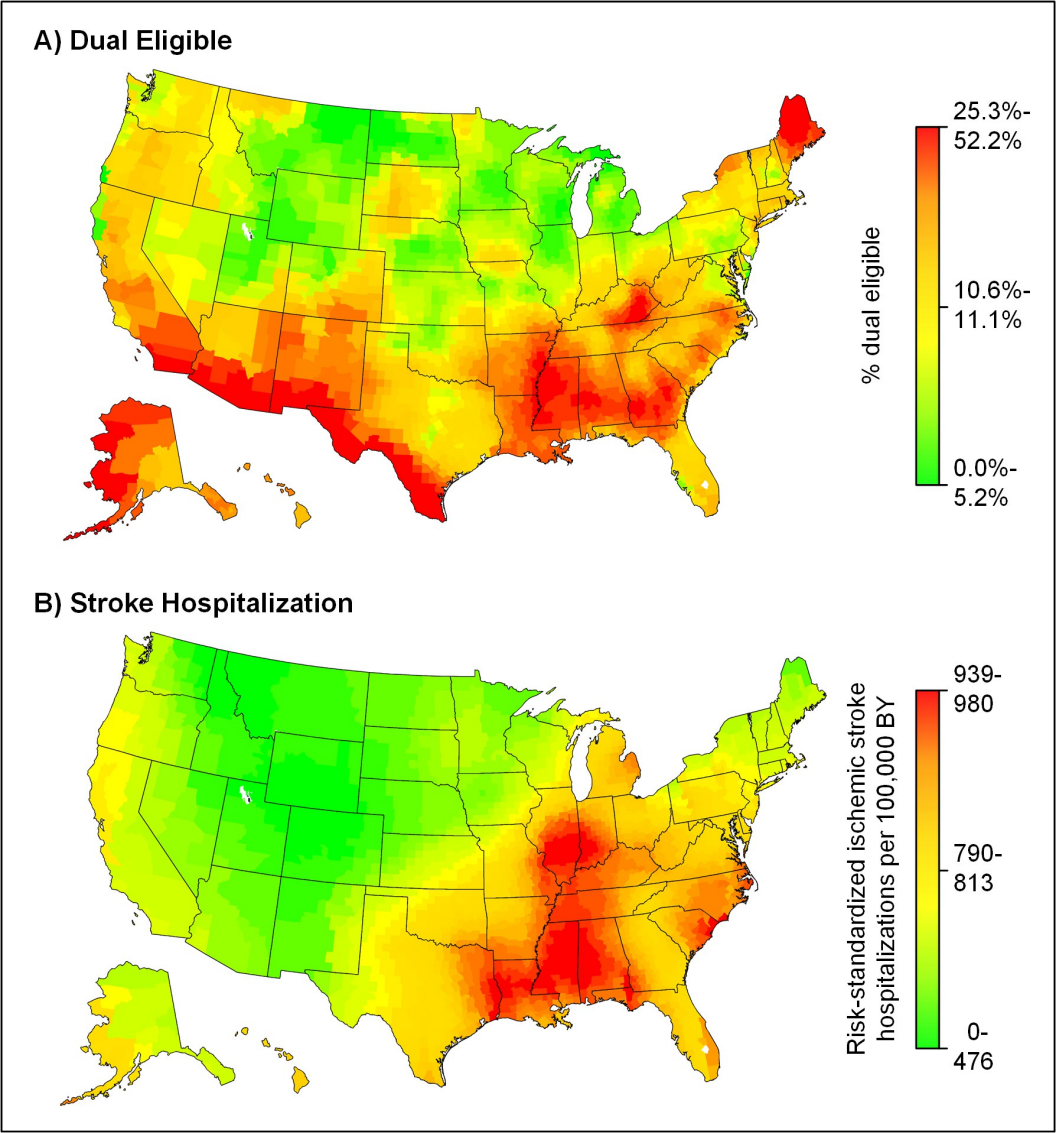
Geographic patterns in dual eligibility and ischemic stroke hospitalization in 2014. A spatial mixed model with a Poisson distribution, county-specific random intercepts, and adjustment for age, sex, race-ethnicity, and Medicare Part B coverage was fit to calculate the risk-standardized rate of ischemic stroke hospitalization for each county. Smoothed rates were obtained for both risk-standardized stroke hospitalization (A) and the percentage of Medicare beneficiaries who were dual eligible (B). Counties or county-equivalents were grouped into 25 quantiles based on these data and shaded according to a gradient from green (lowest percentage dual eligible/lowest hospitalization rate per 100,000 beneficiary-years) to red (highest percentage dual eligible/highest hospitalization rate per 100,000 beneficiary-years). BY, beneficiary-years.

At the national level, 12.5% of all fee-for-service Medicare beneficiaries were dual eligible for Medicare and Medicaid. The highest rates of dual eligibility were in Maine, Alaska, and the southern half of the United States (Fig 1A), whereas the highest rates of stroke hospitalization were concentrated in the central divisions of the South and parts of the Midwest (Fig 1B). There was a moderate correlation between county rates of dual eligibility and risk-standardized stroke hospitalizations per 100,000 beneficiary-years (Pearson’s r = 0.469, p<0.001).

### Stroke patient characteristics and outcome*s*

Among fee-for-service Medicare beneficiaries hospitalized for ischemic stroke, 44,476 were dual eligible (mean age 78.3 years, 64.4% women, 36.4% nonwhite) and 210,426 were Medicare only (mean age 78.9 years, 50.7% women, 11.8% nonwhite) (Table 1). Compared with Medicare-only patients, dual-eligible patients had more comorbidities (e.g., heart failure, renal failure, diabetes), had a longer median length of stay (4 days versus 3 days), had a higher median Medicare payment ($7520 versus $6491), and were more frequently discharged to skilled nursing/intermediate care facilities (32.9% versus 19.2%) (Tables 1 and 2).

**Table 2:** Observed outcomes by dual Medicare-Medicaid-eligible status.

	Dual eligible (n = 44,476)	Medicare only (n = 210,426)	p
**Index hospitalization, median (IQR)**			
Length of stay, days	4 (2–6)	3 (2–5)	<0.001
CMS payment, $	7520 (5444–10634)	6491 (4904–9668)	<0.001
**Discharge disposition, % (95% CI)**			<0.001
Home	24.7 (24.3–25.1)	40.0 (39.8–40.2)	
Home with home health care	12.7 (12.3–13.0)	11.3 (11.2–11.5)	
Skilled nursing/intermediate care facility	32.9 (32.4–33.3)	19.2 (19.0–19.4)	
Inpatient rehabilitation	15.8 (15.4–16.1)	17.1 (16.9–17.2)	
Hospice	4.9 (4.65–5.06)	4.9 (4.79–4.97)	
Transferred	1.9 (1.76–2.02)	1.6 (1.59–1.70)	
**Observed mortality, % (95% CI)**			
In-hospital mortality	4.0 (3.85–4.21)	3.9 (3.86–4.03)	0.437
30-day all-cause mortality	12.4 (12.1–12.7)	11.8 (11.6–11.9)	<0.001
1-year all-cause mortality	28.6 (28.1–29.0)	24.4 (24.3–24.6)	<0.001
**Observed readmission, % (95% CI)**			
30-day all-cause readmission	13.8 (13.4–14.1)	10.8 (10.6–10.9)	<0.001
1-year all-cause readmission	46.7 (46.2–47.2)	39.0 (38.8–39.2)	<0.001

Abbreviations: CI, confidence interval; CMS, Centers for Medicare & Medicaid Services; IQR, interquartile range.

**Fig 2:**
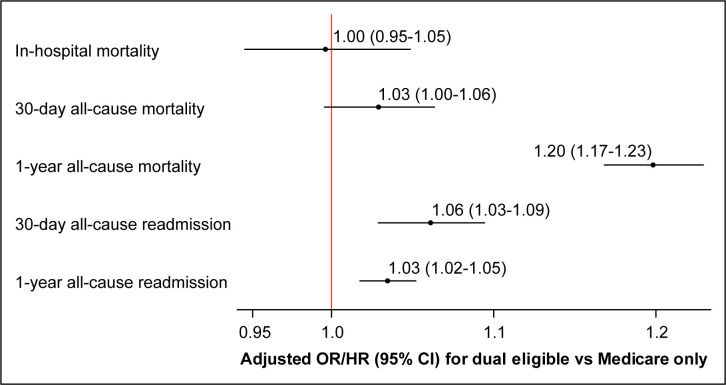
Adjusted associations between dual Medicare-Medicaid-eligible status and outcomes after ischemic stroke. Mixed models with a logit link function were used to calculate odds ratios for the relationship between dual-eligible status and in-hospital, 30-day, and 1-year all-cause mortality. Cox proportional hazards models that censored for change in Medicare enrollment and accounted for death as a competing event were used to calculate hazard ratios for 30-day and 1-year all-cause readmission. All models were adjusted for patient demographics (age, sex, and race-ethnicity) and clinical characteristics (congestive heart failure, prior myocardial infarction, unstable angina, chronic atherosclerosis, hypertension, diabetes, peripheral vascular disease, prior stroke, cerebrovascular disease, renal failure, chronic obstructive pulmonary disease, pneumonia, respiratory failure, cancer, protein-calorie malnutrition, functional disability, dementia, depression, other psychiatric disorder, trauma in past year, and chronic liver disease). CI, confidence interval; HR, hazard ratio; OR, odds ratio.

Dual-eligible patients (versus Medicare-only) had higher observed rates of 30-day (12.4% versus 11.8%) and 1-year (28.6% versus 24.4%) mortality as well as 30-day (13.8% versus 10.8%) and 1-year (46.7% versus 39.0%) readmission; there was no difference in in-hospital mortality (4.0% versus 3.9%) (Table 2). After adjustment, dual-eligible status was associated with greater odds of death within 1 year (odds ratio 1.20, 95% confidence interval [CI] 1.17–1.23) and greater risk of readmission within 30 days (hazard ratio 1.06, 95% CI 1.03–1.09) and 1 year (hazard ratio 1.03, 95% CI 1.02–1.05) (Fig 2).

## Discussion

In this national study of 254,902 fee-for-service Medicare beneficiaries hospitalized with ischemic stroke in 2014, dual Medicare-Medicaid-eligible patients had higher rates of readmission and long-term mortality when compared with Medicare-only patients. These differences persisted even after adjustment for multiple demographic and clinical risk factors. There was no difference, however, in in-hospital or 30-day mortality.

Dual-eligible beneficiaries are among the poorest and sickest in the US healthcare system, and the disproportionate amount of Medicaid and Medicare spending on these patients has garnered increasing attention from researchers and policy makers [8]. There are limited data, however, for stroke. We found that comorbidities and outcomes were worse for dual-eligible versus Medicare-only stroke patients, which is consistent with prior work noting associations between lower socioeconomic status and poor vascular health in terms of risk factors, stroke incidence and mortality, and outcomes after vascular procedures [2,9,10]. Dual-eligible status, however, is more than an indicator of low income among Medicare beneficiaries; it also identifies individuals with additional healthcare coverage through Medicaid. This coverage may include payment of Medicare premiums, Medicare cost sharing, and additional services not covered by Medicare (e.g., transportation to medical appointments, personal care) [8]. Despite these additional benefits, patients in our study had worse post-stroke outcomes. Importantly, we did not find a difference in in-hospital or short-term mortality by dual-eligible status, suggesting that patients who accessed acute care had comparable quality of acute care. However, there may be differences in care at and after discharge contributing to disparities in long-term outcomes.

Although the out-of-pocket cost for care is lowered for those with both Medicare and Medicaid coverage, dual-eligible beneficiaries must navigate two complex programs that are not necessarily aligned regarding financial incentives and patient care. The divided responsibilities between the Medicare and Medicaid programs can result in poor care coordination, communication, and care transitions among providers and healthcare systems, resulting in care that is poor in quality and potentially more expensive [11]. Additionally, patients may not receive all their available benefits. Enhanced discharge planning and improved access to post-discharge resources may be necessary for the vulnerable dual-eligible population to ensure they receive optimal post-stroke care. Additional work is needed to confirm the influence of post-acute care on stroke outcomes for dual-eligible patients as well as to determine the best means of ensuring these patients have the resources they need for recovery.

In addition to assessing differences in outcomes by dual eligibility, we also compared county rates of dual eligibility and stroke hospitalizations among Medicare beneficiaries. We found a moderate correlation between these rates, but there were areas (e.g., the Southwest and Alaska) that had among the highest rates of dual eligibility in the country but lower stroke hospitalization rates. Lower socioeconomic status was associated with stroke incidence in prior work [9]. Our findings suggest that there could be geographic variation, potentially related to differences in population sociodemographic and clinical characteristics, preventive care, or Medicaid eligibility and services that might underlie this observation.

Several limitations should be noted. Our findings are based on Medicare administrative claims data, which are subject to potential coding errors and variation in coding practices across hospitals or geographic regions. Claims data also lack detailed clinical information (e.g., neuroimaging, stroke severity, functional status) that could help further characterize the medical complexity of patients; however, prior research showed comparable performance for claims-based and medical record-based models [12,13]. Because the analyses were limited to fee-for-service Medicare beneficiaries aged 65 years or older, the results may not generalize to younger patients or those without this coverage. Fee-for-service Medicare, however, provides the largest national database of stroke hospitalization rates and post-discharge outcomes and covers the majority of elderly Medicare beneficiaries. Medicaid eligibility, services, and funding vary by state, and we were unable to characterize the specific support provided to patients.

## Conclusion

Dual-eligible stroke patients had higher long-term mortality and all-cause readmission rates than other patients, but there was no difference in short-term mortality. These findings may reflect the poorer health status of dual-eligible beneficiaries but might also be influenced by post-acute care or other social determinants of health. Additional research is needed to better understand the factors contributing to these poorer outcomes for this high-risk population.
